# Potential Role of Inorganic Confined Environments in Prebiotic Phosphorylation

**DOI:** 10.3390/life8010007

**Published:** 2018-03-05

**Authors:** Avinash Vicholous Dass, Maguy Jaber, André Brack, Frédéric Foucher, Terence P. Kee, Thomas Georgelin, Frances Westall

**Affiliations:** 1 Centre de Biophysique Moléculaire (CBM), Centre National de la Recherche Scientifique (CNRS), Rue Charles Sadron, 45071 Orléans, France; avinash-vicholous.dass@cnrs-orleans.fr (A.V.D.); andre.brack@cnrs-orleans.fr (A.B.); frederic.foucher@cnrs.fr (F.F.); 2 Laboratoire d’Archelologie Moléculaire et Structurale, Sorbonne Universités, UPMC Paris 06, CNRS UMR 8220, 4 place Jussieu, F-75005 Paris, France; maguy.jaber@upmc.fr; 3 School of Chemistry, University of Leeds, Woodhouse Lane, Leeds LS2 9JT, UK; t.p.kee@leeds.ac.uk; 4 Laboratoire de Réactivité de Surface, Sorbonne Universités, UPMC Paris 06, CNRS UMR 7197, 4 place Jussieu, F-75005 Paris, France

**Keywords:** nanoscopic confinement, prebiotic chemistry, phosphorylation, hydrogels, interface

## Abstract

A concise outlook on the potential role of confinement in phosphorylation and phosphate condensation pertaining to prebiotic chemistry is presented. Inorganic confinement is a relatively uncharted domain in studies concerning prebiotic chemistry, and even more so in terms of experimentation. However, molecular crowding within confined dimensions is central to the functioning of contemporary biology. There are numerous advantages to confined environments and an attempt to highlight this fact, within this article, has been undertaken, keeping in context the limitations of aqueous phase chemistry in phosphorylation and, to a certain extent, traditional approaches in prebiotic chemistry.

## 1. Introduction

Contemporary cellular systems provide insight into the physicochemical specifics, environment, and the conditions in which the biomolecules efficiently function. In addition to the peculiar nature of water present in these evolved compartmentalized systems [1], we must also take into account the physicochemical environment in which the molecules are dispersed. Under *in vivo* conditions, it is well accepted that crowding (macromolecular crowding in case of cells) is an important phenomenon that contributes to the enhanced functioning of biomolecules in comparison to *in vitro* conditions. Such factors are likely to be overlooked in a typical biochemistry experiment [2]. In the prebiotic milieu there is frequently a focus on the ‘dilution problem’ [3] and molecular crowding within confined dimensions has a role in addressing such an issue. From a chemical point of view, a confined environment provides a kinetic advantage by increasing the effective collision frequency between molecules, which would be a limitation in a dilute environment. The ubiquity of molecular crowding and its consequent effects on reaction rates and equilibrium in cellular entities and biochemistry has been well highlighted by Ellis [2].

The number of exploratory studies in confined systems within the realm of prebiotic chemistry is relatively small. Theoretical studies have been undertaken extensively outside the origins community [2,4,5,6,7,8,9,10,11,12], where the physicochemical properties of water in confinement and its implication on hydrogen bonding have been studied extensively. More recently, within the community, theoretical studies on confinement within layers of mackinawite were carried out where encouraging results have been obtained in terms of amino acid polymerization [13,14,15]. Studies encompassing confinement have been conducted implicitly by others, for example, by Brasier et al. who proposed the pumice hypothesis [16]. This hypothesis is based upon observations of modern pumice rafts, which are colonised by biofilms and organic ‘slicks’. They suggest that pumice can provide ‘natural reaction flasks’, in the form of the ovoid vesicles, for the abiogenic-biogenic transition as it floats on an early Earth hydrothermal ocean. Hazen et al. [17,18,19] have discussed the importance of mineral surfaces, such as rutile, and their role in adsorption, concentration, and chemical reactivity. Westall et al. [20] have emphasized the importance of the sedimentary interface between oceanic crust and seawater and its effects on physical-chemical disequilibria within the interface of porous chemical reactors. On the experimental front, interesting investigations have been undertaken by Hansma (mica hypothesis) [21,22,23,24]. In the mica hypothesis, it is proposed that the interspaces between mica sheets functioned as the primitive cells. Such an environment is considered to have reduced polymer entropy and provided wetting and drying cycles within which prebiotic molecules could potentially evolve, migrate, and ligate to form condensed molecules. Further, it is proposed that mechanical energy (work) could serve as a major energy source to form covalent bonds in such environments. Saladino and co-workers, using zeolite and inorganic catalysts, such as clay and kaolin, demonstrated the synthesis of purine and pyrimidine derivatives in formamide [25]. Zaia emphasized the importance of experiments on inorganic mineral surfaces, chondritic material, and natural minerals for adsorption of amino acids and nucleic acids in seawater [26]. Very recently, phosphorylation in microdroplets was proposed [27,28] and sugar-1-phosphates have been successfully synthesized [29]. However in this particular study, phosphoric acid was used as a phosphorylating agent, whose geological relevance needs to be established. 

Furthermore, some of the aforementioned experiments concern adsorption or catalysis on 2-D surfaces rather than 3-D structures, and none address phosphorylation within confined inorganic frameworks. The ubiquity of phosphorus in biochemical functions, such as replication, metabolism, and, to a certain extent, respiration, is well known [30]. The chemistry of sugar phosphates, in particular, is of critical importance since they are central to understanding the evolution of an ‘RNA world’ that probably preceded modern biochemistry. Energy carriers, such as ATP and ADP, are sugar phosphates that are energy reserves in contemporary cells. The hydrolysis of a condensed phosphate, like ATP, for example, produces −30.5 kJ/mol [30] of Gibbs free energy to perform work. On the primitive Earth, it has been postulated that pyrophosphate [31] or polyphosphates preceded ATP [32], but their formation in the primitive ocean and their lack of stability must be better studied. The free energy associated with such condensed phosphates makes them thermodynamically suitable phoshorylating candidates. Additionally, harvesting of this free energy to useful work is plausible by coupling such hydrolysis to a suitable reaction, for instance, the formation of adenosine monophosphate from phosphoribosyl pyrophosphate [33]. Furthermore, from a biochemical point of view, pyrophosphate and polyphosphates serve as better leaving groups and the free energy of hydrolysis is more valuable from a prebiotic stand-point. Additionally, the phosphoanhydride bonds present in pyrophosphates and polyphosphates are less susceptible to hydrolysis and, thus, relevant in prebiological chemistry. Another advantage of phosphates is their ability to remain charged. The negative charge on a completely deprotonated phosphate is equally spread over the four oxygen atoms giving each P-O a double bond character and the delocalization of the negative charge and pi bond contributes to the overall stabilization. In 1987, Westheimer pointed out the importance of being ionized/charged in “Why Nature chose phosphates” [34,35]. Biomolecules are retained within cell membranes due to their charged nature and this phenomenon most likely played a crucial role on the early Earth. Additionally, the phosphate group is versatile and functions as a buffering agent and chelator of metal ions [35]. Westheimer argued, convincingly, the use of phosphates in current cellular biochemistry and interested readers are directed to [34] and the references therein.

The aim of this paper is to present the advantages of an inorganic confined environment for the phosphorylation processes in order to explain the emergence of a primitive metabolism based on phosphorous chemistry. 

## 2. General Context of Phosphate Condensation

One of the major obstacles in phosphorylation or phosphate condensation is a thermodynamic one [3,36], since it is extremely difficult to remove water between two molecules (to condense) when the two molecules are dispersed in water (Equation (1) Phosphates condensation, Table 1):

HPO_4_^2−^ + HPO_4_^2−^ = P_2_O_7_^4−^ + H_2_O (1) ΔrG° = 10.6 kcal/mol

This problem is fundamental since water was the most abundant solvent on the primitive Earth [37] and, thus, poses a greater challenge in understanding phosphorylation, as phosphorous is quite ubiquitous in contemporary life. Polyphosphates and, to a considerable extent, pyrophosphates, are able to overcome this thermodynamic impediment and could serve as alternatives to orthophosphate compounds in phosphorylation. The chemical potential of these molecules would usually be lost to the environment, but coupling their chemical potential can be directed into prebiotic phosphorylation chemistry. However, an interesting point to note is that, even though the aforementioned condensed inorganic phosphates could have possibly been usable in prebiotic phosphorylation, the synthesis of these molecules as reactants poses exactly the same thermodynamic obstacle, as in the case of any dehydration reaction (Equation (1)). Nevertheless, this is not necessarily a problem in the case of an open system, where water or polyphosphate can be removed, thus preventing the system from attaining chemical equilibrium. Table 1 summarizes the free enthalpy of some phosphorylated molecules. 

These facts compelled researchers to look for alternatives, such as mineral surfaces, to overcome the thermodynamic and kinetic barrier [39]. Minerals have been found to have a stabilizing effect on certain molecules and are able to immobilize organic molecules on their surface [39,40]. Since control of water activity/low-water activity aids in overcoming the thermodynamic problem, minerals have reached the forefront of prebiotic chemistry research. For a detailed account of the history of phosphorylation in prebiotic chemistry, readers are directed to [30,35,41] and the references therein, since the subject falls beyond the scope of the discussion at hand. More recently, Gull et al. [36] demonstrated the successful phosphorylation of nucleosides adenosine and uridine in the presence of the meteoritic analogue of the mineral schreibersite under mild environmental conditions. Moreover, a one-pot synthesis of phosphoribosyl pyrophosphate (PRPP) was recently demonstrated for the canonical nucleotide adenosine monophosphate from its building blocks (KH_2_PO_4_ or Pi, adenine, and d-ribose) on a fumed silica surface [33]. In considering all of these experiments, the geological relevance of the minerals and their probable availability on the early Earth needs to be taken into consideration. Even though mineral surfaces have advantages over aqueous environments in terms of adsorption and concentration, in the formation of long-chain polymers (by dry-wet cycling), the interaction of the latter with the surface becomes much stronger and desorption is more difficult [42]. Enthalpy of desorption is, therefore, much greater. Given the high negative charge present on phosphates and polymers involving phosphates, this would be an important consequence to consider on phosphorylation involving mineral surfaces. Such a scenario would be under control in the case of a confined-hydrated environment due to diffusivity and yet maintaining low-water activity.

## 3. Anomalous Water Properties in Confined Environments

Given the constraints associated with phosphorylation and, especially, the thermodynamic constraint due to water, it compels us to investigate alternatives to lower this energy barrier or get around this thermodynamic barrier. More recent studies on water in a confined environment are emerging as promising avenues and could well aid in overcoming this thermodynamic impediment. The following is a brief discussion on the effects of water in confined environments.

**Figure 1:**
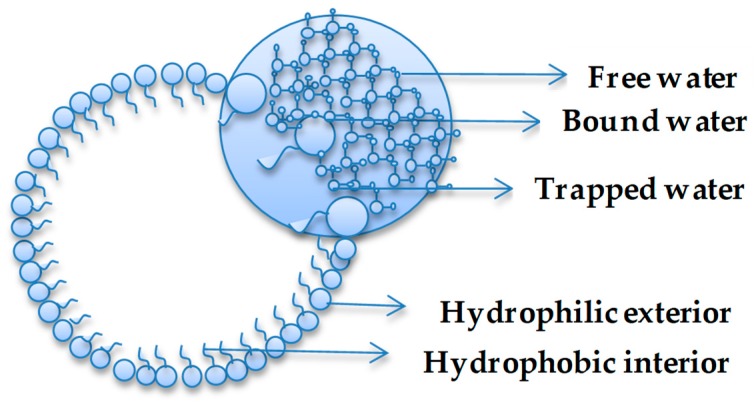
Water types in confined system.

Environment plays a critical role in shaping the macroscopic properties of a system. Similarly, the environment, including confinement, plays a role in determining the behaviour of water [4,8,10]. Water confinement is of great interest to biology, geology, materials science, nanoscience, and recent studies in prebiotic chemistry are proving the importance of understanding the nature of water in restrained spaces [15]. Venables et al. [5] have provided clear evidence that water shows anomalous hydrogen bonding properties as a function of size, as demonstrated, for example, in reverse micelle (RM) systems. The studies documented that interfacial interactions were magnified as a function of micelle size when a nanopool of water was dispersed within a system characterized by a hydrophilic interior and hydrophobic exterior (i.e., reverse micelle). In confined environments, the H-bonding behaviour of water is linked to a two-state system model [5] or a three-state system model [43], which are often termed as ‘types’ of water. In the two-state system, water is considered to be ‘bound or free’ based on the extent of hydrogen bonding [43,44,45] and this has been extended to ‘trapped’ water in a three-state system, where the water is trapped between hydrophilic sites [43]. Figure 1 illustrates the types of water present in a general hydrophilic-hydrophobic confinement system. The same scheme could be used to illustrate the states of water in inorganic structures, such as silica.

Venables and co-workers investigated the changes arising in the absorption band at 670 cm^−1^ from vibrational motion of the bonded hydrogen atom. This band is characterized by the motion of the OH group and is considered sensitive to the environment of hydrogen-bonding. They also pointed out that, even though the -OH stretching band could indicate weak hydrogen bonding, the signal arising from a librational band was far more sensitive for studying hydrogen-bonding in the smallest of micelles.

Such studies indicate that there exists a spectrum, of varying extent, in hydrogen bonding, which persists only up to a few molecular layers beyond which the bulk water properties take effect. Experimental results have revealed that smaller and intermediate micelles with a diameter (*d*) ranging from 2 to 5 nm show pronounced interfacial effects in comparison to large micelles (*d* ≥ 5 nm) where the bulk-water properties are predominant [10]. Some experimental results demonstrate that, regardless of the nature of the interface, nanoscopic confinement has a major impact on water hydrogen bond network dynamics [8]. This hydrogen bonding environment of water is important to control the overall water activity and presumably has an impact upon phosphorylation and other prebiotically-relevant processes in confined environments. Additionally, the effect of water monolayers with molecules close to the interfaces is considerably affected and is explained later in this section by electrical double-layer influences. 

Another consequence attributed to a confined environment is its effect on the dielectric constant of interfacial water in comparison to bulk water, where charged species are stabilized to a greater extent at the interfacial region in comparison to bulk-water [46]. This conforms to Davis’ principle- the importance of being charged [34], observed at a lower confinement scale. This also allows for the restriction of water and its solutes to the hydrophilic regions and prevents out-diffusion of the solutes to the external environment (dilution of components is an extended consequence). Confinement is also known to have a profound effect on the diffusivity of solvents. This was demonstrated in a molecular dynamics study of acetonitrile in pores with hydrophilic silanol (-OH) terminations and pores with hydrophobic terminations [12]. From these studies, it was concluded that the diffusion of acetonitrile occurred irrespective of the type of hydrophilic or hydrophobic termination, but that diffusivity was lowered in the presence of silanol terminations. It was also reported that the slower diffusivity along the axis (*z*) of a hydrophilic pore was a factor ~0.7 slower in comparison to the hydrophobic pore. Radial diffusion (*x*, *y*) was reported to be unaffected, in general (a factor of ~3.3 slower than the one-dimensional diffusion in the bulk liquid; for the hydrophobic pore, by a factor of ~2.6 in comparison to the bulk liquid). The dielectric constant [14] and diffusivity [47] of water in confined environments are understood to partly contribute to altering the energetics and mechanism of reactions in comparison to reactions in bulk water [15] and such effects are theoretically applicable in the case of phosphorylation as well. 

Another interesting aspect of confined water is the efficient proton transfer at extreme confinement (by the Grotthuss mechanism) by retaining this property from bulk water [46,48]. Such efficient proton transfer in confinement might have preceded the efficient proton channels that are observed in contemporary cells and maintain a natural proton gradient. Further interesting studies have revealed that, even at below-freezing temperatures, confined adsorbed water in 1 nm pores is mobile in comparison to water confined in 10 nm pores [49]. The authors of this study propose that this phenomenon is subject to efficient proton exchange and hydrogen bond rearrangement of adsorbed water. Such localities should be sites of interest for prebiotic chemistry, where the interplay of relatively low temperature and organic molecule stability within a low-water activity environment could serve an important role. Proton transfer is vital to contemporary life and natural proton gradients are well known to aid ADP to ATP conversion [50,51] and a primitive form of proton motive force could have existed on early Earth, which evolved into a complex energy transducer of the present day.

Another anomalous behaviour of water was studied by Kolesnikov and co-workers [52] by neutron scattering studies and *ab initio* simulations, demonstrating a new quantum tunnelling state of water by confinement inside the mineral beryl (beryllium aluminium cyclosilicate, Be_3_Al_2_Si_6_O_18_). Tunnelling is a quantum-scale phenomenon where a particle overcomes an energy barrier, which is forbidden in classical mechanics. The authors noted that protons of water in the tunnelling state occupy six symmetrically-equivalent diverse positions, each with a 50 meV energy barrier and its dipole moment is therefore modified by this tunnelling. Among the six tunnelling orientations possible, one of the orientations would result in an orientation where the protons are directionally opposite to each other and the resulting net dipole moment is equal to zero. Theoretically, this will have consequences whereby a momentary loss of polarity of the water molecule will result in water behaving as a non-polar solvent. Such a phenomenon, where the polarity of water fluctuates between polar and non-polar states will have implications for prebiotic chemistry as water can potentially be lipophillic momentarily. In such a scenario, water will be able to aggregate polar molecules, such as sugars and, also, the sluggish aggregation of lipids, giving rise to a gradually self-organised system.

Interface chemistry is classically understood in terms of a virtual electric double layer. A solid-liquid interface, such as mineral-water, is also explained by a double-layer model. Mineral-water interfaces are considered high electric field environments [53]. In the thesis study by Laporte, an attempt to understand the electric field contribution of a surface to the free energy landscape of prebiotic reaction was tested [53]. It was observed that non-polar surfaces, such as MgO, exhibit high electric fields at distances of up to 3 Å. When monolayers of water molecules are added, the zone of the high field seems to be available to molecules about 5 Å away from the original surface. In a way, this first layer of water serves to spread the high-field interface. Part of the study suggests that local electric fields at the surface of dry-wet oxides and minerals are sufficiently intense to modify the free energy landscape of surface chemical reactions. The ramification of such high energy fields (>10^−1^ V/Å) [54] could be harvested in phosphorylation and prebiotic chemistry in general. Electric fields on scales of V/Å are equivalent to those experienced by valence electrons of atoms and molecules [54]. This could easily allow redistribution of valence electrons and, thus, influence the molecular layers adsorbed on the surface of minerals and the molecules diffusing in proximity of such high field interfaces. For example, based on Coulomb’s law, localized charges in zeolite cavities are estimated to have fields of the order 1 V/Å [54].

## 4. Effect of Confinement on Energetics

Munoz-Santiburcio and Marx [46] have recently reported *ab initio* studies on peptide synthesis (by activation via N-carboxyanhydride) and their investigations of interfacial water between iron-sulphur minerals have revealed that charged species and transition states required lower activation energy in comparison to bulk water and water in extreme conditions. They attributed this observation to the enhanced dielectric properties of water in confinement, where the charged species are stabilized and the zwitterionic forms are prevalent, which, in turn, reinforces the importance of charged species in present-day cellular compartments. The significance of phosphorylation under hydrated conditions and its relevance has been briefly outlined in the Section 2. Theoretical work using statistical–mechanical principles have revealed exceptional differences in chemical equilibrium in confinement when compared to macroscopic systems [11]. Here, the equilibrium constant was observed to considerably favour products as an inverse function of size. Polak and Rubinovich [11] accredited this observation to reduction of the number of microstates in a reaction mixture and their virtual isolation from the immediate surroundings. This is true for any such pore system as the ‘effective collision frequency’ is increased (not neglecting the stoichiometry of the reactants). This should be theoretically appropriate for phosphorylation (dehydration reactions) in hydrated environments with reduced water activity and anomalous water properties. Arguments along similar lines were put forth by Hansma [23], where it was reasoned that the shorter distances between molecules in confinement would enhance reaction yields (a possible consequence of chemical equilibrium). The mica hypothesis proposes that confinement between mineral sheets is a form of entropy reduction [21] in an open system and this point has been emphasized implicitly in numerous works on the subject of molecular confinement and in prebiotic chemistry. The diffusivity of a solvent in a porous system is also understood to be a function of increased activation energy of diffusion near hydrophilic surfaces and, thus, reduced diffusivity of the solvent in comparison to bulk [12]. This diffusive activation energy is more convoluted and is a result of the interplay of several factors simultaneously whose overall effect is nevertheless of relevance to prebiotic chemistry in confinement.

## 5. Geological Environment: Organic Versus Inorganic

In terms of the chemistry of the origins of life (abiogenesis), the inclusion of prebiotic reactions pertinent to the early Earth demands adherence to geologically-relevant environments. In the 1990s, it was postulated that the first step toward the origin of life was the spontaneous condensation of amphiphilic molecules (chemical compounds which are both hydrophilic and lipophilic) to form vesicles [55]. In addition, examples of autocatalytic micelle growth have been noted [56]. However, the physical and thermal stability of such systems needs to be considered in the context of the early geological conditions that prevailed on the early Earth. The extreme sensitivity of such systems to fluctuations in temperature, pH, pressure, mechanical shock, or other environmental factors, could have played a major role in shaping proto-life and the subsequent evolution to contemporary life. The Hadean Eon (the time period between the accretion of the Earth to ca. 4 Ga), during which life arose on Earth, was a considerably different world to the one on which we now live [57,58,59]. Atmospheric [56,57] and oceanic differences [60,61], primitive planetary dynamics [58,62], and lithologies unlike those currently able to form on the planet point towards an Earth that cannot be closely approximated by the modern Earth.

Inorganic systems, on the other hand, were undoubtedly an integral part of the Hadean Earth. A series of studies by Hazen and collaborators has addressed the issue of chemistry at geological interfaces, proposing that crystal surfaces of Hadean Eon-relevant minerals provide an effective substrate upon which the concentration of prebiotic molecules could have been achieved [17,18,19]. These works have demonstrated the pivotal role played by mineral-water interfaces in both concentration and catalytic processes [39,40]. This approach provides a valuable direction for necessary future studies to further elucidate the importance of mineral surfaces at the prebiotic-biotic transition.

**Figure 2:**
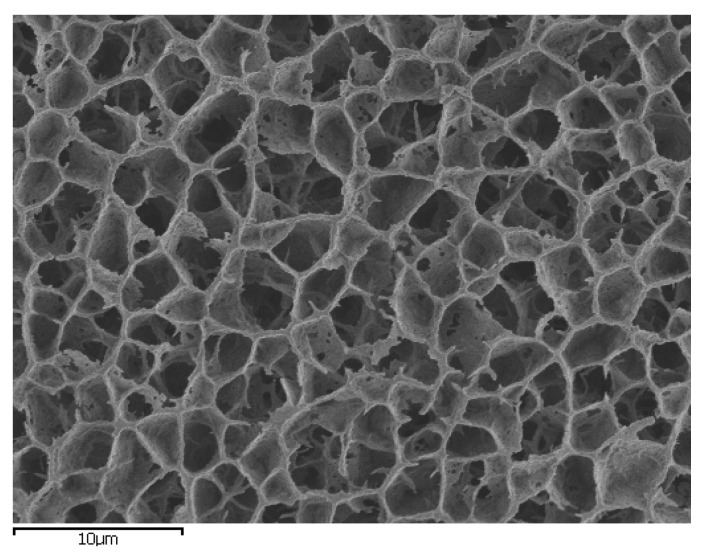
Cryosem image of silica hydrogel prepared by a sol/gel methods from a mixture of sodium silicate and acetic acid solutions. Pore size around 2 µm.

Keeping this in mind, we reiterate an experimental suggestion initiated some years ago, which is yet to make significant inroads within abiogenesis and, which would have significant potential to influence the ‘problem of phosphorus and phosphorylation’. Whilst most prebiotic-related experiments are conducted within an aqueous solvent matrix composed of dissolved salts and contiguous mineral surfaces, Trevors and Pollack pointed out that contemporary biochemistry does not function within such a matrix, but one (the cellular cytosol) that more closely resembles a heterogeneous hydrogel [1,63]. They propose that experimental prebiotic studies should benefit from being performed in such a medium [1,47]. Mineral hydrogels are, we believe, a geologically-plausible mechanism to maintain concentration gradients, alter the structure of water, and influence ion-macromolecule interactions [47]. Gradients of ions (protons and metal cations) would be one of the defining features of inorganic hydrogels since their ability to exclude solutes [47,1] will maintain the overall system out of equilibrium by creating an imbalance of solutes inside and outside the gel and, thus, power the system to remain in a state of ‘life’. Hydrogels have significant value as proto-cytosolic media wherein the need for a fragile amphiphilic cell membrane or an organic medium is obviated. As this domain of prebiotic chemistry evolves, so should our approach since such a 3-D environment has the possibility of acquiring compositional and pH gradients that are different to those of its immediate surroundings. This is because such a porous media (e.g., Figure 2), analogous to a membrane, is known for the selective and nonselective exchange of ions and, thus, maintenance of a charge difference that, in turn, could be harnessed as an electrical potential [64]. Furthermore, an interesting trait of interfacial or encapsulated water is that solutes are excluded as a function of their size [1]. Considering the enthalpic, entropic, and physical constraints, preferential solute exclusion tends to be a predominant phenomenon and the larger solutes tend to avoid the structured water environment inside a porous gel [1,47,65,66]. An analogous solute segregation study, with respect to size, was demonstrated in gels at distances in the order of 100 μm or greater [67]. Hydrogels with their low-water activity are, thus, capable of hosting condensation reactions, such as phosphorylation/polymerisation, as well as readily absorbing and concentrating ionic components of interest, hence, preventing their loss into the surrounding environment [1,47,68]. The phase transition of a gel is a dynamic process, where a significant amount of work occurs [69], resulting in a high degree of volume [1,70] and structural changes [1]. The ensuing domino effect gives rise to desiccated, hydrated zones within the gel polymeric network. Such a locality of low-water activity is best suited for phosphorylation and condensation reactions in prebiotic chemistry. Taking a cue from nature, studies by Baltscheffsky et al. with certain bacteria [31] have shown that pyrophosphate aided growth [35] and some microbes harvested sunlight to photophosphorylate, thus producing pyrophosphate rather than ATP [31,35]. Hydrogels may well possibly harvest sunlight to photophosphorylate while partially shielding the interior components from intense radiation, subject to the thickness of the gel. Non-enzymatic pyrophosphate synthesis under visible light was already proposed during the 1980s [71]. Such stellar intrusion could also serve a secondary function, where the exterior of the gel is slowly dehydrated, thus driving water from the interior of the hydrogel toward the exterior or, in certain cases, such as silica hydrogels, desiccation creates a hydrophobic exterior which traps the internal components within. 

Systems which act in response to stimuli are interesting candidates for investigations in prebiotic chemistry. Stimuli-responsive polymer gels that are sensitive to their surroundings, such as temperature, pH, electric field, and light, are not unfamiliar in the domain of hydrogel chemistry [70,72]. Given the geological relevance of inorganic-hydrogels [20,63], and their response to stimuli, such as light and temperature, it is tempting to suggest an analogy between inorganic hydrogels and the most primitive, non-enzymatic stimuli-responsive abiogenic cells having rudimentary cellular capabilities and capable of reproducing solely by means of mechanical/physical shocks, similar to seed dispersal. 

## 6. Conclusions

Confined environments have great potential in phosphorylation chemistry and prebiotic chemistry, in general. Given the thermodynamic barrier that exists within phosphorylation chemistry in bulk water, confinement offers the ability to explore environments that have a positive impact on the physicochemical nature of the water within. The ramifications of such anomalous behaviour of water allows for controlled diffusivity, low-water activity and, more importantly, opens up alternate avenues in terms of thermodynamic and kinetic manoeuvrability. Furthermore, we propose an inorganic confined environment as the next logical step for phosphorylation chemistry, keeping the geological relevance in mind. 

## Figures and Tables

**Table 1:** Free enthalpy of phosphorylated molecules, from orthophosphates [38].

Molecules	∆rG° (kJ/mol)
Pyrophosphate	+42
Adenosine-monophosphate	+183
Adenosine-diphosphate	+279
Adenosine-triphosphate	+550
